# New tubular injury markers in children with a solitary functioning kidney

**DOI:** 10.1007/s00467-014-2802-y

**Published:** 2014-03-22

**Authors:** Katarzyna Taranta-Janusz, Beata Zalewska-Szajda, Elżbieta Gościk, Sylwia Chojnowska, Małgorzata Dmochowska, Marta Pszczółkowska, Anna Wasilewska

**Affiliations:** 1 Department of Pediatrics and Nephrology, Medical University of Białystok, 15-274 Białystok, Waszyngtona 17 Poland; 2 Department of Imaging Diagnostics, Medical University of Białystok, Children Hospital, Białystok, Poland; 3 Medical Institute, College of Computer Science and Business Administration of Lomza, Lomza, Poland

**Keywords:** Exoglycosidases, Solitary functioning kidney, Tubular damage

## Abstract

**Background:**

The present study aimed to assess whether the urinary profiles of the lysosomal exoglycosidases N‑acetyl‑β‑hexosaminidase (HEX) and its isoenzymes A (HEX A) and B (HEX B), α-fucosidase (FUC), β-galactosidase (GAL), α-mannosidase (MAN), and β- glucuronidase (GLU) are useful biomarkers of tubular dysfunction in children with a solitary functioning kidney (SFK).

**Methods:**

We measured the urinary activity of HEX, its isoenzymes HEX A, HEX B, and FUC, GAL, MAN, and GLU in 52 patients with SFK. Patients were subdivided into two groups: congenital SFK (cSFK)—unilateral renal agenesis and acquired SFK (aSFK)—unilateral nephrectomy. The reference group (RG) contained 60 healthy sex- and age-matched children.

**Results:**

Urinary activity of all exoglycosidases in SFK was significantly higher than in RG (*p* < 0.05). There were no differences in exoglycosidase activity between cSFK and aSFK (*p* > 0.05). HEX and its isoenzymes HEX A and HEX B correlated negatively with estimated glomerular filtration rate (eGFR), and all estimated parameters correlated positively with albumin/creatinine ratio (*p* < 0.001).

**Conclusion:**

Urinary activity of HEX, its isoenzymes HEX A and HEX B, and FUC, GAL, MAN, and GLU is elevated in children with SFK. Long-term follow-up studies in larger groups of children with SFK may help us to better understand their clinical significance.

## Introduction

Routine ultrasound screening of pregnant women has led to an increase in the number of detected cases of a solitary functioning kidney (SFK). General incidence of SFK is 1 in 2,000 people [1]. Although some authors consider SFK to be a harmless condition, there are reports in the literature that have demonstrated that even 40–50 % of adults with SFK require dialysis [2].

Children with a congenital solitary functioning kidney (cSFK) are expected to develop compensatory glomerular hyperfiltration during childhood [3]. Studies in diabetes have demonstrated that glomerular enlargement is associated with hypertrophy of tubules [4]. This process mainly affects the proximal tubular cells. Experimental studies in animal models suggested that in diabetic kidney, proximal tubular hypertrophy might play a central role in the pathogenesis of glomerular hyperfiltration [4]. Vallon et al. [5] presented data obtained in a murine diabetic model suggesting that proximal tubular growth was necessary to allow enhanced proximal tubular reabsorption with consequent glomerular hyperfiltration.

Cells of the renal epithelium synthesize and excrete many enzymes to the urine. Determination of enzyme activities in urine is a sensitive and non-invasive method for the evaluation of renal tubular function.

Exoglycosidases are hydrolases involved in post-translational modifications of glycoproteins and degradation of glycoconjugates (glycoproteins, glycolipids, and proteoglycans). A gradual release of terminal sugars from glycoconjugate oligosaccharide chains in lysosomes is observed [6]. Exoglycosidases can be released to the cell cytosol, then out of the cell to the blood stream and urine, as a result of the increased permeability of lysosomes caused by disease progression [7]. As all glycoconjugates have N-acetylhexosamines, HEX (N-acetyl-β-hexosaminidase) is a universal marker for the degradation of glycoconjugates. A lack or a significant decrease in activity of a lysosomal enzyme in tissues and body fluids results in lysosomal storage disease.

In the urine of healthy individuals, activity of N-acetyl-β-hexosaminidase (HEX) is negligible, but significantly increases in response to proximal tubular damage [8]. Renal proximal tubule epithelial cells are very sensitive to hypoxia. Therefore, all renal processes involving hypoxia lead to dysfunction of proximal renal tubules and release HEX into the urine. Increased activity of HEX in urine has been found because of heavy metal intoxication [9], nephrotoxic drugs [10], fever [11], nephritis [12], hypertension [13], diabetes [14], neoplasms [15], and during renal graft rejection [16]. Other lysosomal exoglycosidase activity, i.e., α-fucosidase (FUC), β-galactosidase (GAL), α-mannosidase (MAN), and β-glucuronidase (GLU), have been evaluated in numerous physiopathological situations such as hepatocellular carcinoma (HCC), liver disease, and gastric tumors, alcohol dependence, and colon and larynx cancers [17–22], but not in kidney diseases in children.

So far, however, there has been little discussion about the role of lysosomal exoglycosidases in children with SFK.

The aim of this preliminary study was to evaluate the activity of N‑acetyl‑β‑hexosaminidase (HEX), its isoenzymes A (HEX A) and B (HEX B), α-fucosidase (FUC), β-galactosidase (GAL), α-mannosidase (MAN), and β-glucuronidase (GLU) in order to verify urinary lysosomal exoglycosidase usefulness as a marker of proximal tubular damage in patients with SFK.

## Patients and methods

This cross-sectional study included all patients with SFK who were referred to the Department of Pediatrics and Nephrology Medical University of Bialystok, Poland, for diagnostic work-up or control evaluation between January 2012 and March 2013. Fifty-two patients (32 male and 20 female, aged median 9.75 year Q1–Q3: 4.5–15.0) with SFK documented by renal ultrasound and renal scintigraphy, were divided into two groups according to whether they had a congenital (cSFK) or an acquired solitary functioning kidney (aSFK).

Participants were required to meet the following inclusion criteria: age 2 months to 18 years; presence of SFK that was normal on imaging; cSFK or aSFK. The following exclusion criteria were used: detectable ultrasound abnormalities (scarring and/or hydronephrosis) of a single kidney; history of urinary tract infection prior to investigation, and taking any medication that might influence renal function.

The reference group (RG) consisted of 60 healthy participants with a pair of normal kidneys visualized by ultrasound, who were offspring of the Department’s employees and healthy school pupils recruited from the OLAF study [23]. Children were age- and gender-matched (36 male, 24 female, aged median 10.03 years Q1–Q3: 8.02–13.54). They were considered “healthy” if clinical history, physical examination, urine test, and renal ultrasound were normal.

Demographic and clinical data were assessed. In all children, careful clinical history, underlying comorbidities, and physical examination were estimated. Body mass index (BMI) was calculated as weight (kg) divided by the square of height (m^2^). BMI Z-scores, which reflect the SD score for age- and gender-appropriate BMI distribution, were calculated using the formula: Z = [X − μ]/σ; where X is the BMI measured in the patient, and μ and σ represent the mean and standard deviation for age- and gender-matched healthy children [24]. Age- and height-specific reference values for BMI and height were generated using the LMS method [25]. Blood pressure (BP) was measured in the sitting position with the use of a sphygmomanometer with the appropriate cuff using the standard method. The average of the three readings was calculated. Glomerular filtration rate (GFR) was assessed by endogenous creatinine clearance (C_cr)_ and updated Schwartz formula – eGFR = 0.413x [height in cm/sCr] [26]. It is known that the Schwartz formula clearly overestimates C_cr_ in the majority of cases. [27]. We did not use renal inulin clearance, the gold standard for GFR, because it suffers from a number of pitfalls, i.e., lack of availability, difficult assays, and problems with collecting timed urine samples, especially in children who have not yet mastered toilet training. The kidney overgrowth (O %) was calculated for every child, according to the formula and with reference to the charts [28]: O % = ((individual renal length − 50th centile of renal length for age, gender, and side of the body)/individual renal length) × 100 %.

All urine specimens were collected from morning samples, centrifuged, and supernatants were frozen at −80 °C until further analysis. Assays were performed within 3 months. Urinalysis was done for all patients. Urinary albumin and creatinine were measured in morning samples, and the urinary albumin/creatinine ratio (ACR) was calculated. Urine albumin concentration was determined by Lowry’s method and the results were expressed in μg/mL. Children and adolescents with a urinary albumin/creatinine ratio between 30 to 300 μg/mg were considered to have microalbuminuria.

The activity of HEX, its isoenzymes HEX A, HEX B, and of FUC, GAL, MAN, and GLU in urine (pKat/mL) was determined using the method described by Marciniak et al. [29] and modified by Szajda et al. [30]. To 10 μL of appropriately diluted urine were added 40 μL of 0.1 M phosphate-citrate buffer at pH 4.7 for HEX and pH 4.3 for the remaining exoglycosidases, and 30 μL of 20 mM substrate solution of p-nitrophenyl-N-acetyl-ß-D-glucosaminide for the determination of HEX; p-nitrophenyl-α-L-fucopyranoside for FUC; p-nitrophenyl-β-D-galactopyranoside for GAL; and p-nitrophenyl-α-D-mannopyranoside for MAN (Sigma, St. Louis, MO, USA). The mixtures were incubated for 60 min at a temperature of 37 °C. The reactions were stopped by adding 200 μL of 0.2 M borate buffer at pH 9.8. The activity of lysosomal exoglycosidases corresponding to the amounts of released p-nitrophenol were measured at 405 nm using a microplate reader ELx800 and KC junior computer program (Bio-Tek instruments, Winooski, VT, USA). The concentration of activity of lysosomal exoglycosidases was expressed in pKat/mL. Urinary creatinine concentrations were used to adjust the urinary activity of HEX, its isoenzymes HEX A, HEX B, and of FUC, GAL, MAN, and GLU.

After informed written consent was secured from the parents of the patients and reference group, kidney size and function were evaluated according to the standard protocol approved by the Bioethics Committee of the Medical University of Białystok, Poland, in accordance with the Declaration of Helsinki.

Computer program Statistica version 10.0 (StatSoft, Tulsa, OK, USA) was used for statistical analyses. Adequacy of parameters for normal distribution was tested using the Shapiro–Wilk test. Significant differences were compared using the Mann–Whitney and Wilcoxon tests for independent and dependent variables. Correlations between variables were evaluated using Pearson‘s or Spearman’s test as appropriate. A *p* value <0.05 was considered significant. Receiver operating characteristic (ROC) curves were used to determine the cut-off values of urinary exoglycosidase activity that gave the best sensitivity and specificity.

## Results

The clinical characteristics of patients compared with healthy children are summarized in Table 1. The overall median age of all children with SFK was 9.75 years. The age and gender of the children examined did not differ from the reference group (*p* > 0.05). Boys predominated, as well as SFK on the left side (57.69 %). Of 52 patients eligible for analysis, 41 children (78.8 %) had cSFK and 11 (21.2 %) had aSFK. Children with cSFK were younger than those with aSFK (*p* < 0.05). Mean systolic and diastolic blood pressure measurements in both subgroups of SFK were within the normal ranges. The etiologies of the aSFK were as follows: ureteropelvic junction obstruction with/without ureteral or bladder abnormalities (36.36 %), ureterovesical junction obstruction (45.45 %), and reflux nephropathy (18.9 %). At the time of nephrectomy, all children had normal contralateral kidneys on ultrasound and functional imaging. None of our patients had proteinuria in their morning sample. Microalbuminuria was diagnosed in 30 patients with SFK.

**Table 1:** Characteristics of patients (with a solitary functioning kidney [*SFK*]) and the reference group (*RG*)

		SFK	RG	*p*
		Median (Q1–Q3)		
Age (years)		9.75 (4.5–15.0)	10.03 (8.02–13.54)	NS
Height (cm)		142 (112.5–164.0)	141.6 (130.4–161.0)	NS
Weight (kg)		31.0 (20.0–60.5)	34.77 (28.6–51.55)	NS
BMI (Z–score)		0.4 (−0.29 to 1.0)	0.37 (−0.22 to 0.93)	NS
Blood pressure	Systolic (mmHg)	105 (87–130)	101 (82–126)	NS
	Diastolic (mmHg)	68 (40–94)	62 (49–91)	NS
O%		17.8 (11.06–24.71)	–	–
eGFR (mL/min/1.73 m^2^)		106.04 (93.32–127.07)	–	–
Albuminuria (μg/mL)		69.72 (35.07–115.45)	47.9 (36.26–72.66)	NS
Albumin/creatinine ratio (μg/mg)		69.19 (44.17–137.7)	44.07 (35.15–57.02)	<0.01

*BMI* body mass index, *eGFR* estimated glomerular filtration rate, *O%* kidney overgrowth, *Q1* lower quartile, *Q3* upper quartile, *NS* not significant

As shown in Table 2, the urinary activities of all exoglycosidases expressed both as pKat/mL and adjusted to creatinine were significantly higher in SFK children than in healthy participants (*p* < 0.05). When compared with the reference group, cSFK patients had significantly higher values of activity for all exoglycosidases (*p* < 0.05). Similar results were found in aSFK children, excluding HEX A (*p* > 0.05). On the other hand, there was no difference in HEX, HEX A, HEX B, FUC, GAL, MAN, and GLU urinary activity between cSFK and aSFK patients (*p* > 0.05). There was no correlation of estimated parameters with age (*p* > 0.05). Urinary albumin/creatinine ratio was significantly higher in the examined group of children than in the reference group (*p* < 0.01).

**Table 2:** Estimated markers in children with a solitary functioning kidney (*SFK*) and the reference group (*RG*); comparison of the results with reference group

Estimated markers	SFK	RG	*p*
	Median (Q1–Q3)		
HEX (pKat/mL)	167.89 (130.2–235.74)	117.24 (94.1–165.22)	<0.01
HEX A (pKat/mL)	27.64 (10.05–83.55)	19.75 (9.59–29.91)	<0.05
HEX B (pKat/mL)	129.57 (106.95–174.8)	90.15 (73.78–119.5)	<0.01
FUC (pKat/mL)	119.52 (93.13–136.48)	76.04 (63.05–91.84)	<0.01
GAL (pKat/mL)	111.35 (84.96–172.92)	82.25 (68.13–96.92)	<0.01
MAN (pKat/mL)	111.35 (82.45–131.45)	81.12 (67.57–93.53)	<0.01
GLU (pKat/mL)	130.82 (104.44–157.21)	78.29 (63.62–106.5)	<0.01
HEX (pKat/μg Cr)	0.19 (0.13–0.49)	0.09 (0.07–0.16)	<0.01
HEX A (pKat/μg Cr)	0.03 (0.01–0.14)	0.01 (0.007–0.03)	<0.01
HEX B (pKat/μg Cr)	0.15 (0.1–0.29)	0.07 (0.05–0.13)	<0.01
FUC (pKat/μg Cr)	0.12 (0.08–0.23)	0.07 (0.04–0.09)	<0.01
GAL (pKat/μg Cr)	0.12 (0.08–0.27)	0.07 (0.05–0.1)	<0.01
MAN (pKat/μg Cr)	0.11 (0.07–0.24)	0.07 (0.04–0.1)	<0.01
GLU (pKat/μg Cr)	0.15 (0.08–0.28)	0.08 (0.04–0.1)	<0.01

*HEX* N‑acetyl‑β‑D‑hexosaminidase, *HEX A* isoenzyme A, *HEX B* isoenzyme B, *FUC* α‑fucosidase, *GAL* β‑D‑galactosidase, *MAN* α‑mannosidase, *GLU* β-glucuronidase

Median eGFR was 106.04 mL/min/1.73 m^2^, and correlated negatively with age in SFK patients (r = −0.33, *p* < 0.01). A negative correlation was found between HEX, its isoenzymes HEX A, HEX B (pKat/mL), and eGFR (r = −0.29, r = −0.25, r = −0.29; *p* < 0.05 respectively) in children with SFK. No correlations between the urinary activity of estimated markers expressed as pKat/μg Cr and serum creatinine, eGFR, were found. It is noteworthy that the SFK group showed very strong positive correlations between the urinary activity of all estimated exoglycosidases (pKat/μg Cr) and albumin/creatinine ratio (*p* < 0.001; Table 3).

**Table 3:** Statistically significant correlations between estimated markers and albumin/creatinine ratio (*ACR*) in solitary functioning kidney children (Spearman’s correlation analysis)

	ACR	r	*p*
HEX/Cr		0.55	0.00002
HEX A/Cr		0.46	0.0005
HEX B/Cr		0.53	0.00003
FUC/Cr		0.51	0.00008
GAL/Cr		0.49	0.0001
MAN/Cr		0.51	0.0001
GLU/Cr		0.54	0.00003

Furthermore, neither the urinary-specific activity of HEX and its isoenzymes HEX A, HEX B, nor FUC, GAL, MAN, and GLU (pKat/mL) activity correlated with the compensatory overgrowth of the kidney (O%). Similar results were obtained for the urinary activity of measured exoglycosidases corrected for urinary creatinine. Kidney overgrowth (O%) did not correlate with eGFR and ACR.

As shown in Table 4, ROC analyses were performed in order to define the diagnostic profile of estimated exoglycosidases in identifying children with early signs of renal damage (presence of microalbuminuria) among patients with SFK. In this analysis the AUCs for HEX and its isoenzymes HEX A, HEX B, and for FUC, GAL, MAN, and GLU were 0.95, 0.815, 0.91, 0.8, 0.885, 0.83, and 0.87 respectively.

**Table 4:** Receiver operating characteristic (ROC) analyses for urinary activity of HEX, its isoenzymes HEX A, HEX B, and of FUC, GAL, MAN, and GLU (pKat/Cr) in the study group (SFK)

pKat/μg Cr	AUC	SE
HEX	0.95	0.035
HEX A	0.815	0.112
HEX B	0.91	0.061
FUC	0.8	0.122
GAL	0.885	0.066
MAN	0.83	0.102
GLU	0.87	0.076

## Discussion

The most interesting findings of our study concerning exoglycosidases in children with SFK are as follows:

1. Urinary activity of all exoglycosidases expressed as pKat/mL and adjusted to creatinine in children with SFK were significantly higher than in healthy patients
2. There was no statistically significant difference between cSFK and aSFK
3. Very strong positive correlations were found between the urinary activity of all estimated exoglycosidases adjusted to creatinine and the urinary albumin/creatinine ratio
4. ROC analyses showed good diagnostic profiles for all estimated exoglycosidases in identifying children with early signs of renal damage (presence of microalbuminuria) among SFK children

The present study was designed to determine whether in children with SFK the early stage of renal injury is observed and if the urinary activity of exoglycosidases might be useful as potential biomarkers of this injury.

Data from the literature indicate that GFR may remain constant in the early stages of chronic kidney disease, whereas the nephron endowment deteriorates secondary to a renal disease; thus, patients with a normal GFR may or may not be hyperfiltrating [31]. In our study, only 2 patients revealed hyperfiltration. However, it is noteworthy that the age of the study participants ranged from 2 months to 18 years.

In this current report 30 out of 52 children had albuminuria. The urinary albumin/creatinine ratio was significantly higher in the group of children who had been examined than in the reference group.

The results of this study may indicate that in children with SFK, tubular injury is present. It is confirmed by increased activity of all the lysosomal enzymes examined. A recent hypothesis suggested by Thomson and colleagues [4] proposed that proximal tubular hypertrophy might be the primary event, driving activation of the renin–angiotensin–aldosterone system (RAAS) and glomerular hyperfiltration. A potential mechanism exists for the relationship between glomerular hyperfiltration and the increase in kidney volume observed in patients with SFK. Specifically, renal RAAS activity is stimulated in SFK and increased levels of angiotensin II may cause both glomerular hyperfiltration and inflammation, oxidant injury, and fibrosis of kidney tissue.

Experimental studies have shown that reduction of renal mass causes hyperfiltration, a compensatory mechanism that prevents GFR decline. The long-term consequences of this state are proteinuria, hypertension, and a decrease in the GFR. It is considered that albuminuria represents a predominantly hemodynamic effect of glomerular hypertension and hyperfiltration [32]. A significant proportion of patients with cSFK develop albuminuria, which may be interpreted as a sign of hyperfiltration. People with cSFK and elevated urinary albumin excretion may be at increased risk of declined renal function.

During the past decades, it has become more clear that proteinuria itself can play a pathogenic role, thereby contributing to tubulo-interstitial damage. Different theories exist regarding the potential mechanisms of proteinuria-induced tubular cell injury. The excessive reabsorption of ultrafiltered proteins by proximal tubular cells can lead to tubular damage and apoptosis/necrosis by exhaustion of the lysosomal degradation pathway and spillage of lysosomal enzymes into the cytoplasm. In response to excessive lysosomal protein degradation, the proximal tubular cells produce a variety of molecules, including lysosomal exoglycosidases.

Until a few years ago, the almost exclusive clinical application of the assay of HEX isoenzymes was the biochemical diagnosis of gangliosidoses and the detection of carriers [33]. However, more recently, numerous studies have reported that HEX and its isoenzyme HEX B may be markers of cell damage [34], and they have become prominent in the identification of renal injury [35].

Our results confirm the hypothesis that tubular injury might be an important component of SFK damage. It is worth mentioning that there was a very strong correlation between the urinary albumin/creatinine ratio and enzyme activity expressed in pKat/Cr.

Similar findings were noted in proteinuric patients. Patients with tubular proteinuria showed a significant increase in β-hexosaminidase only at the acute stage of the renal disease, while patients with chronic tubular dysfunction did not show any increase in β-hexosaminidase excretion [36]. With this data, we can address the question of whether HEX and/or its isoenzymes in urine directly reflect tubular cell damage or merely increased turnover. Our study suggests that the urinary activities of HEX and its isoenzymes A and B might serve as useful markers of renal damage in SFK. This hypothesis should be confirmed in further studies because elevation in the urinary activity of exoglycosidases may be not only a result of proximal tubule injury, but also of enhanced lysosomal activity without injury per se [37].

Other estimated markers were α-fucosidase (FUC), β-galactosidase (GAL), α-mannosidase (MAN), and β-glucuronidase (GLU) enzymes, which play a key role in carcinogenesis. FUC is a glycosidase involved in the metabolism of certain glycolipids and glycoproteins, identified as a novel and promising marker of cellular senescence. GAL is classical marker that is widely used to detect senescent cells. Lysosomal MAN plays a vital role in maintaining cellular homeostasis. GLU is an exoglycosidase involved in stepwise degradation of glucuronic acid-containing glycosaminoglycans. Our present data revealed higher FUC, GAL, MAN, and GLU activity in children with cSFK and aSFK than in healthy participants. We did not find any studies analyzing FUC, GAL, MAN, and GLU activity in children with renal disease. On the other hand, alterations in the activity of FUC, GAL, MAN, and GLU enzymes have been described in different human malignancies [17, 19, 38–40]. In addition, some studies have reported that α-mannosidase activity might be a useful marker of *Chlamydia trachomatis* in urogenital tract specimens [41]. Significant increases in the activity of HEX A, GAL, and FUC were reported in the saliva of patients with HIV infection, as well as a significant decrease in the activity of HEX B [42].

An additional interesting finding in our study was the negative correlation of the urinary activity of exoglycosidases (pKat/Cr) and eGFR with patient’s age. This is in agreement with the observations of Wikstad et al. [43] and Baudoin et al. [44], who noticed that in adults with SFK since childhood renal function tends to decrease slowly but significantly.

Increases in the activity of exoglycosidases in bodily fluids may be inexpensive and sensitive markers for the preliminary screening of many diseases. Activities of exoglycosidases in bodily fluids could be especially valuable in monitoring chronic diseases, as urine can be obtained using non-invasive methods.

These observations have led to the proposal that urinary activity of estimated exoglycosidases adjusted to creatinine can be considered to be potentially useful, non-invasive markers of kidney damage in patients with SFK. This proposal is supported by data from our ROC analyses, which showed good diagnostic profiles for all the exoglycosidases assessed.

We believe that an increase in urinary enzymes precedes clinical symptoms and is a more sensitive diagnostic marker than microalbuminuria. However, our study has a few limitations. This is a single-center study that included only 56 patients. Therefore, further large-scale studies should be conducted to extend our results to other populations. Long-term follow-up studies in adults are warranted as well, to determine whether HEX and its isoenzymes A and B are useful as new tubular markers in the prediction of renal function decline. Finally, evaluation of these biomarkers in children with different stages of chronic kidney disease (CKD) are needed, as well as studies predicting renal prognosis in different patients with CKD.

## Conclusions

The urinary activity of HEX, its isoenzymes HEX A, HEX B, and FUC, GAL, MAN, and GLU is elevated in children with SFK. A combination of urinary exoglycosidase levels has an additive clinical value for the prediction of kidney injury. Long-term follow-up studies in larger groups of children with SFK may help us to better understand their clinical significance.
